# A Review and In Silico Analysis of Tissue and Exosomal Circular RNAs: Opportunities and Challenges in Thyroid Cancer

**DOI:** 10.3390/cancers14194728

**Published:** 2022-09-28

**Authors:** Eman A. Toraih, Mohammad H. Hussein, Manal S. Fawzy, Emad Kandil

**Affiliations:** 1 Division of Endocrine and Oncologic Surgery, Department of Surgery, School of Medicine, Tulane University, New Orleans, LA 70112, USA; 2 Genetics Unit, Department of Histology and Cell Biology, Suez Canal University, Ismailia 41522, Egypt; 3 Department of Medical Biochemistry and Molecular Biology, Faculty of Medicine, Suez Canal University, Ismailia 41522, Egypt; 4 Department of Biochemistry, Faculty of Medicine, Northern Border University, Arar 91431, Saudi Arabia

**Keywords:** circular RNAs, circRNA, diagnosis, exosomal circRNAs, functional enrichment analysis, genome-wide circRNAs, online databases, prognosis, treatment resistance

## Abstract

**Simple Summary:**

Thyroid cancer is the most common endocrine neoplasm. Recently, knowledge of the molecular genetic changes of thyroid cancer has dramatically improved. Understanding the roles of these molecular changes in thyroid cancer tumorigenesis and progression is essential in developing a successful treatment strategy and improving disease outcomes. As a family of non-coding RNAs, circular RNAs (circRNAs) have been involved in several aspects of the physiological and pathological processes of the cells. The roles of circRNAs in cancer development and progress are evident. In the current review, we aimed to explore the clinical potential of circRNAs as potential diagnostic, prognostic, and therapeutic targets in thyroid cancer. Furthermore, screening the genome-wide circRNAs and performing functional enrichment analyses for all associated dysregulated circRNAs in thyroid cancer have been done. Given the unique advantages circRNAs have, such as superior stability, higher abundance, and presence in different body fluids, this family of non-coding RNAs could be promising diagnostic and prognostic biomarkers and potential therapeutic targets for thyroid cancer.

**Abstract:**

Thyroid cancer (TC) is the most common endocrine tumor. The genetic and epigenetic molecular alterations of TC have become more evident in recent years. However, a deeper understanding of the roles these molecular changes play in TC tumorigenesis and progression is essential in developing a successful treatment strategy and improving patients’ prognoses. Circular RNAs (circRNAs), a family of non-coding RNAs, have been implicated in several aspects of carcinogenesis in multiple cancers, including TC. In the current review, we aimed to explore the clinical potential of circRNAs as putative diagnostic, prognostic, and therapeutic targets in TC. The current analyses, including genome-wide circRNA screening and functional enrichment for all deregulated circRNA expression signatures, show that circRNAs display atypical contributions, such as sponging for microRNAs, regulating transcription and translation processes, and decoying for proteins. Given their exceptional clinical advantages, such as higher stability, wider abundance, and occurrence in several body fluids, circRNAs are promising prognostic and theranostic biomarkers for TC.

## 1. Introduction

Thyroid cancer (TC) is the most prevalent endocrine-related neoplasm, and its incidence continues to increase [1]. Papillary thyroid carcinoma (PTC) is the most common type of thyroid cancer, accounting for nearly 85% of cases [2]. Although the five-year overall survival rate of patients with PTC is nearly 95%, the cancer can metastasize into lymph nodes and distant organs, resulting in poor prognosis and recurrence in nearly 30% of patients [3]. It is critical that we unravel the mechanisms underlying cancer initiation and progression in order to design novel diagnostics and therapeutics.

Non-coding RNAs (ncRNAs) are non-protein coding transcripts that can be classified into two main types: small ncRNAs (18–200 nucleotides) and long ncRNAs (lncRNAs, >200 nucleotides) [4,5]. The microRNA family (miRNAs; 22–25 nucleotides) belongs to the former class, binding to miRNA response elements (MREs) in the 3′-untranslated region (3′-UTR) of the target messenger RNA (mRNA) to induce mRNA degradation and/or suppress its translation into protein [6,7].

Circular RNAs (circRNAs) have emerged as a large family of endogenous ncRNA transcripts widely expressed in different cellular contexts and can act as post-transcriptional regulators. They are generated by head-to-tail splicing that has neither 5′–3′ polarities nor a polyadenylated tail, thus exhibiting a covalently closed loop structure [8,9]. Most circRNAs are derived from the gene’s exon region, but a small percentage are created by intron cleavage [10]. They were first identified in humans nearly 40 years ago using electron microscopy [8]. Initially, circRNAs were misinterpreted as splicing errors and transcriptional artifacts [11,12,13]. Given the recent advances in high-throughput sequencing and bioinformatics analyses, thousands of circRNAs have been recognized as abundant stable ncRNAs in various species and cell lines [14].

Several studies have emphasized the essential roles of circRNAs in both normal cellular processes and pathological conditions, as depicted in diverse types of cancers [15,16,17,18,19]. Various circRNAs have been recognized as cancer-specific non-coding RNAs in tumorigenesis [20], such as circMTO1 in hepatocellular carcinoma, CCDC66 in colon cancer, hsa_circ_0067934 in esophageal squamous carcinoma, circAGFG1 in breast cancer, and cTFRC in bladder carcinoma [14,21,22,23,24,25,26]. The aberrant expression of circRNAs may impact their regulatory roles in gene expression, which contributes to carcinogenesis by disrupting several signaling pathways, including the Wnt/β-catenin pathway [27], phosphatidylinositol-3-kinase (PI3K)/AKT signaling transduction pathway [28], vascular endothelial growth factor A (VEGFA)/VEGF receptor-2 pathway, and Ras/extracellular signal-regulated kinase (ERK) cascade signaling pathway [29]. These pathways control multiple cellular processes that drive cancer development, including cell growth, cell cycle, migration, angiogenesis, survival, and apoptosis, among others.

A growing body of literature supports the association of circRNAs with PTC carcinogenesis [27,30,31,32]; however, the exact role of circRNA in thyroid tumorigenesis and progression has not been fully clarified. Overall, the current review aims to explore the clinical utility of circRNAs as putative prognostic and theranostic targets in TC by revising related information from the published literature on several data sources and running in silico analysis to provide a suggested list of differentially expressed circRNAs in thyroid cancer.

Throughout this review, a brief presentation of the circRNA nomenclature, biogenesis, biological activities, and the online related databases/resources has been carried out, followed by a review of the diagnostic and prognostic utility of circRNAs in patients with TC, how the circRNAs mediate tumor progression in vivo and in vitro, the tumor-cell-derived exosomal circRNAs, their implication in treatment resistance, functional enrichment for their deregulation, screening the genome-wide circular RNAs and identifying their microarray transcriptomic signature in PTC, and lastly, a discussion of the current challenges in clinical practice and future perspectives have been provided.

### 1.1. CircRNA Nomenclature

There are no specified HUGO standards for gene nomenclature for circRNAs, but recent guidelines recommend using “circ(gene symbol)-n”, where the gene symbol represents the unspliced “host” gene and n is a five-digit number listing the order of discovery for circRNAs [33]. For example, the 15 circRNAs that are derived from the *EGFR* gene are named “hsa_circEGFR_001” to “hsa_circEGFR_015” [20] with “hsa (homo sapiens)” indicating human-derived transcripts.

### 1.2. CircRNA Biogenesis, Export, and Turnover

**Figure 1:**
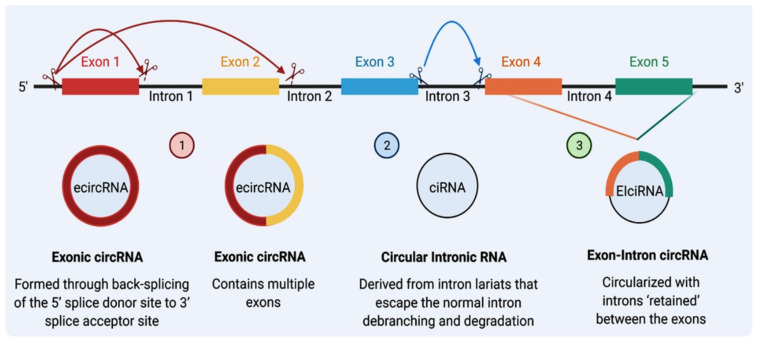
The biogenesis of circRNAs. There are three major classes of circRNAs: Class (1) Exonic circular RNA (ecircRNA): Initially, intron No. 1 is removed, and the 5′ splice site of exon No. 2 is brought close to 3′ splice site of Exon No. 1, creating ecircRNA that contains multiple exons. Exons can also skip splicing; exon No. 1 can also link with exon No. 3. Class (2) Circular intronic RNA (ciRNA): Reverse complementary sequences of lariat intron spliced from pre-mRNA can pair to produce a closed-loop structure termed ciRNA. Class (3) Exon–intron circRNAs (EIciRNAs): Intron No. 4 can be retained with Exon No. 4 and Exon No. 5 to form an EIciRNA (Created by Biorender.com).

CircRNA and linear RNA are usually are co-expressed [20]; however, nascent circRNAs are generally processed at a later stage than linear ones [34]. The size of a circRNA molecule can range from <100 to >4000 nucleotides [35]. They can be formed from spliced introns or ≥1 exons [36]. Based on their origin from different regions of the genome, circRNAs can be classified into three types: (1) exonic circRNA (EcircRNA), representing the majority of circRNAs (more than 80%), is derived from direct back-splicing or exon skipping; (2) circular intronic circRNAs (ciRNA) that consist of lariat introns that are generated due to the presence of specific elements near the branchpoint site and the 5’ splice site, these types of introns are much more likely to contain complementary inverted Alu repeats, which is the most common transposon in the genome [35]; and (3) exon–intron circRNA (EIciRNA) that contains both exons and introns [15,35,37] and is produced from exons, introns, or both [36,38] (Figure 1). Two other types of circRNAs, intergenic and antisense, are uncommon and remain poorly understood [39].

Several types of circRNAs are created from precursor mRNAs (pre-mRNAs) by distinct processes such as exon skipping, intron pairing, and RNA-binding proteins [15,39,40]. Given the endless nature of circRNAs, this adds exceptional stability and resistance to enzymatic digestion by exonucleases [20,35] and allows some circRNAs to accumulate to levels that may surpass the linear forms [41]. CircRNA half-lives can reach up to 48 h, relative to about 10 h for linear RNAs [35]. Rates of biogenesis, cellular export, and turnover, together regulate the steady-state intracellular levels of circRNAs [42]. EcircRNAs are predominantly found in the cytoplasm, whereas ciRNAs and EIciRNAs are more abundant in the nucleus [38]. How circRNAs are exported from the nucleus is not entirely clear, but the N6-methyladenosine (m6A) modification is often found in circRNAs and has been shown to affect their nuclear export [43]. CircRNAs were also found in secreted extracellular vesicles and were more abundant in exosomes than cancer cell lines [44,45,46].

CircRNAs often have tissue-restricted and cancer-specific expression profiles, which are regulated by specific trans-acting factors and cis-acting elements. They can accumulate to levels exceeding messenger RNAs and show cell/development-specific expression. In cancer, multiple factors can affect the balance between canonical RNA splicing for intron removal between adjacent exons, and back-splicing, leading to deregulation of circRNA expression [42]. The elucidation of deregulated circRNAs and identification of their biological roles are ongoing processes in cancer research.

### 1.3. Biological Activity of circRNAs

**Figure 2:**
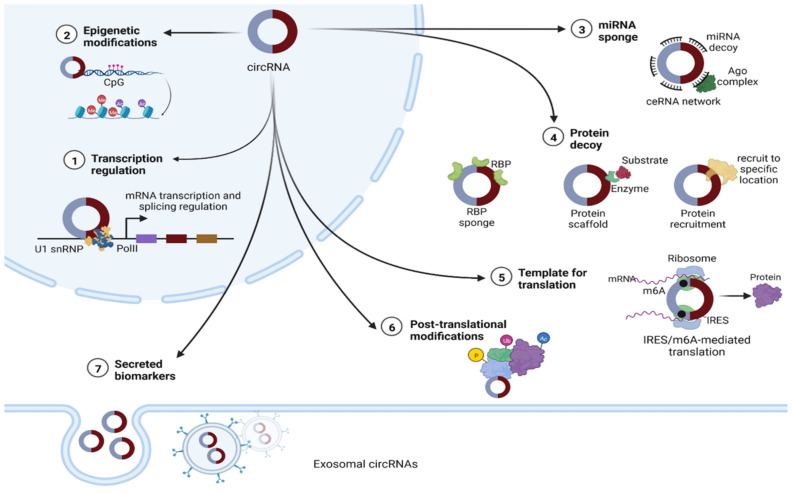
The putative functions of circRNAs. (1) The nuclear type can act as regulators of their host genes’ transcription, either by interacting with U1 small nuclear ribonucleoprotein (U1 snRNP) and enhancing RNA polymerase II (Pol II) function, or by recruiting methyl cytosine dioxygenase (TET1) to the promoter sequences [51]. (2) Epigenetic regulation, including DNA and histone modifications [51]. (3) In cytoplasm, circRNAs can act as microRNA sponges, leading to a disrupted competing endogenous RNA (ceRNA) network [16,50]. (4) CircRNAs can function as RNA binding protein (RBP) decoys or otherwise can act as modulators for specific RBPs’ half-life, promoting or reducing their degradation by proteasomes. Enabling the colocalization of enzymes to their substrates facilitates reaction kinetics. CircRNAs can also act as protein scaffolds. (5) CircRNAs with internal ribosome entry site (IRES) elements and the initial methionine code (AUG) for translation sites can be translated into proteins through a CAP-independent mechanism, facilitated by the presence of methyl adenosine (m6A) and other translation-specific factors. (6) CircRNAs have been implicated in several posttranslational modifications, including phosphorylation (P), ubiquitylation (Ub) and acetylation (Ac). (7) Finally, circRNAs can be secreted in extracellular vesicles regulating intercellular communication [56].

There is growing evidence that circRNAs can influence a range of biological pathways through diverse mechanisms of action, including serving as microRNA sponges [47,48,49], protein decoys [16,50], regulators of transcriptional activity [51] that can mediate the parental expression genes [52], regulators of alternative splicing [16], and modulators of mRNA stability [53]. In addition, circRNAs can act as templates for protein translation and sequester proteins from their indigenous subcellular localization [16,50,51,54] (Figure 2). CircRNAs are also known to function as mediators of RNA–protein interactions [55] and scaffolds for protein complex assembly [9].

Nuclear circRNAs have been implicated in regulation of transcription via protein recruitment or as structural scaffolds. Cytoplasmic circRNAs function in several ways, including as miRNA sponges and/or RNA-binding proteins and as scaffolds for proteins or enzyme–substrate complex assembly, facilitating posttranslational modifications [20]. CircRNAs have also recently been identified as essential components of the microvesicle cargo and the exosome, where they can be enriched related to their cell production level [56].

#### 1.3.1. Epigenetic Regulators

A handful of circRNAs can indirectly affect transcription by modulating histone modification via miRNA binding. For example, circ_100284 is involved in malignant transformation via microRNA-217 regulation of “enhancer of zeste homolog 2 (EZH2)” [57], whereas circ_0071589 can intensify the expression of EZH2 methyltransferase through sponging miR-600 [52].

Furthermore, circRNAs can mediate epigenetic modification through DNA methylation. For example, circ-FECR1 can directly bind to the promoter of DNMT1 methyltransferase, leading to transcription inhibition [58].

#### 1.3.2. Transcription Regulators

Several circRNAs remain at their nuclear sites of synthesis and seem to function as gene expression regulators for their parent coding genes [15]. Exonic circRNAs, which make up the majority of the explored circRNAs, are mainly located in the cytoplasm; therefore, they are unlikely to play a role in regulating the transcription process [59]. On the other hand, two EIciRNAs, circ-EIF3J and circ-PAIP2, were found to bind with U1 snRNP, forming a complex with RNA Pol II in the promoter sequence of parental genes, boosting their transcription through a positive feedback loop [52]. In addition, other circular introns have the potential to bind to the elongating RNA Pol II and enhance the transcription of their host gene [60]. Studies have also suggested that circRNAs can act as splicing regulatory factor processors, since the back-splicing mechanism during which they are formed competes for precursor mRNA with the canonical mechanism of linear RNAs [16,61].

#### 1.3.3. Regulation of Transcription Factors

Emerging evidence shows that circRNAs regulate the activity of the transcriptional activators and/or repressors activity that control the expression of key players in tumor biology. The circular form of TP53 (circ-TP53) was found to inhibit the linear p53 transcription factor [30]. The basic helix-loop-helix transcription factors, TCF3 and TCF4, were downregulated by circ_102171 in TC [27]. The same circular RNA can suppress the DNA-binding transcription factor LEF1 [27], whereas circ-ABCB10 represses the expression level of the C2H2 zinc finger transcription factor KLF6 [47]. On the other hand, circ-FAT1(e2) causes overexpression of ZEB1, a C2H2 zinc finger transcription factor, via its sponging of miR-873 [62]. Circ_0001018 and circ_0005273 are involved in mediating the expression of the HMG box transcription factors SOX2 and SOX4 [63,64]. Similarly, circ-NRIP1 can directly interact with miR-195-5p to regulate the levels of the DNA-binding transcription factor STAT1 [65], and circ-PRKCI is involved in regulating E2F3 by directly binding to miR-335 [66]. Moreover, circ_0039411 promotes the expression of the MTA1 transcription factor via sponging miR-1179 and miR-1205 [49]. Given their role as transcriptional regulators, circRNAs are related to the expression of target miRNAs and proteins associated with TC cell proliferation, invasion, and metastasis, contributing to TC development (Table 1).

**Table 1:** Transcriptional regulation by circRNAs in thyroid cancer.

Transcription Factors	Type of TF	circRNA	Parental Gene	miRNA Sponge	Reference
Upregulated TF					
↑MTA1	Homeodomain TF	circ_0039411		↓miR-1179, miR-1205	[49]
↑ZEB1	C2H2 zinc finger TF	circ-FAT1(e2)	*FATI*	↓miR-873	[62]
↑SOX2	HMG box TF	circ_0005273		↓miR-1138	[64]
↑SOX4	HMG box TF	circ_0001018		↓ miR-338-3p	[63]
↑STAT1	DNA-binding TF	circ-NRIP1	*NRIP1*	↓miR-195-5p	[65]
↑E2F3	General TF	circ-PRKCI	*PRKCI*	↓miR-335	[66]
Downregulated TF					
↓TCF3, TCF4, LEF1	Basic helix-loop-helix TF	circ_102171			[27]
↓TP53	P53-like TF	circ-TP53	*TP53*	↓miR-1233-3p	[30]
↓KLF6	C2H2 zinc finger TF	circ-ABCB10	*ABCB10*		[47]

#### 1.3.4. Acting as Molecular Sponges

RNA transcripts such as mRNAs, lncRNAs, and pseudogenes engage in mutual regulations with each other via competitively shared miRNAs [14]. The latter can downregulate gene expression, destabilize target RNAs, or inhibit translation [67]. In this way, depending on the target genes, miRNAs can act as oncogenes or tumor-suppressor genes. It has been speculated that all classes of RNAs may compete for miRNAs. This competing endogenous RNA (ceRNA) activity forms wide-scale trans-regulatory crosstalk across the transcriptome and plays essential roles in cancer [68].

It has been shown that circRNAs are an essential subtype of ceRNAs. They can act as miRNA molecular sponges via binding to miRNA response elements (MREs), attenuating the effect of miRNAs on target gene expression [50]. CircRNAs can also contain multiple MREs that regulate gene expression at the transcriptional and/or post-transcriptional level [50]. Because circular RNA sponges are characterized by high expression levels, stability, and many miRNA binding sites in their structure, they are likely to be more effective sponges than linear transcripts [69].

**Figure 3:**
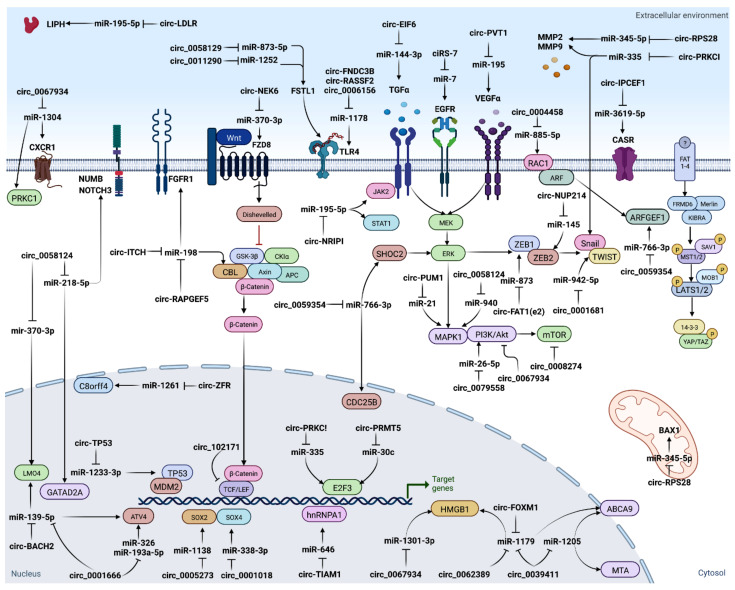
CircRNAs serve as miRNA sponges.

The “circRNA-miRNA-target gene” axis could play an essential role in regulation of several biological features of cancers. It has been found that numerous ceRNA networks are dysregulated during TC tumorigenesis, invasion/metastasis, epithelial–mesenchymal transition (EMT), and therapeutic resistance [70] (Figure 3). Many studies have suggested that circRNAs function as miRNA sponges, with subsequent loss of miRNA suppressive function on target genes. For example, circ-ITCH inhibits TC progression by sponging miR-22-3p and upregulating CBL level [71]; circ_0025033 accelerates TC proliferation through sponging miR-1233, and miR-1304 [48]; circ_0005273 acts as an oncogene through targeting miR-1138 [64]; circITGA7 acts as miR-198 ceRNA to regulate FGFR1 expression [72]; and overexpression of circular RNA circ-NEK6 abrogates the tumor suppressive effect of miR-370-3p on TC via the WNT signaling pathway [73]. Since deregulation of the ceRNAs networks is implicated in cancer initiation and progression, it can provide an opportunity for targeted therapeutics.

#### 1.3.5. Acting as Regulators of Translation into Protein

Due to their lack of polarity, IRES, and 5′ cap, circRNAs were first thought to be incapable of forming proteins; however, evidence shows that some circRNAs can act as templates for protein translation due to harboring initiating sequences [61,74,75]. For example, “circ-SHPRH” can encode the SHPRH-146 amino acid protein, which acts as a tumor suppressor [76].

#### 1.3.6. Acting as Protein Decoys

Beyond regulating microRNAs, circular RNAs may bind and sequester RNA-binding proteins (RBP) or even base pair with RNAs besides microRNAs, forming large RNA–protein complexes [77]. For example, ciRS-7 is a novel circRNA that regulates miRNA, mRNA, and RBP functions to influence specific physiological processes [77]. As an oncogene, ciRS-7 suppresses miR-7 and promotes tumor growth in several cancer types. Furthermore, circRNA ciRS-7 has been implicated in promoting EMT in PTC cells [78], and via modulating the miR-7/EGFR axis, ciRS-7 enhances PTC cell proliferation and migration cells [78]. Circ-PABPN1 binds to ELAV-like protein 1, one of the RBPs, preventing its binding to its cognate PABPN1 mRNA with subsequent PABPN1 translation suppression [79]. Circ_102171 activates the Wnt signaling pathway by binding to CTNNBIPI [27].

### 1.4. Online Databases and Resources

Multiple websites and freely accessible circRNA-related online databases have recently been established. For example, “CircAtlas v2.0, CIRCpedia v2, and TCSD” provide the expression levels of circRNA across different human tissues/cell lines. Also, “CircRiC” and “MiOncoCirc v2.0” explore circRNAs in cancer cell lines and human cancer samples, respectively. “CircAtlas v2.0, circBase, and circRNADisease”, in addition, present circRNA–miRNA interactions. “CircInteractome, CircRiC, CSCD, TSCD, CircFunBase, and Circ2Disease” predict “circRNA–RBP” interactions. Meanwhile, “CIRCpedia, circFunbase, Circad, NPInter (v4), and CircAtlas (v2)” databases cover circRNA-related information across different species. The complete list of these databases [80,81,82,83,84,85,86,87,88,89,90,91,92,93,94,95,96,97,98,99,100,101,102,103,104,105,106,107] with their description and weblinks were detailed in our previous work [108].

## 2. Evidence Acquisition

This study provides a review of 64 related published original articles in PubMed and Google Scholar databases through 23 June 2022, and by applying keywords such as “(circular OR circRNA) AND (thyroid) AND (cancer OR carcinoma OR tumor)” to explore the role of circular RNAs in thyroid tumorigenesis and progression in humans, and navigate the in vitro and in vivo studies to assess their diagnostic/prognostic performance as tissue and liquid biopsy biomarkers. References of all relevant review articles have also been cross-checked against the search.

## 3. Results

This section of the current review includes six subsections; the diagnostic and prognostic utility of circRNAs in TC patients, circRNAs mediation for tumor progression in vivo and in vitro, tumor-cell-derived exosomal circRNAs, circRNAs and treatment resistance, functional enrichment analysis for the deregulated markers, and genome-wide circular RNA screening.

### 3.1. Diagnostic and Prognostic Utility of circRNAs in TC Patients

In the last decade, circRNAs have received much attention as key players in cancer development and progression, supporting their utility as biomarkers for cancer diagnosis or monitoring [42]. Because of their closed-loop structure, circRNAs have a high level of stability and resistance to degradation [20,35] and are thus likely to be promising biomarkers for PTC. Many circRNAs show increased expression levels in TC tissues and cell lines compared with adjacent non-cancer tissues, normal tissues, and normal cell lines and promote tumor development.

The “circRNA pleckstrin and Sec7 domain containing 3 (circ_PSD3)” were aberrantly upregulated in PTC tumor tissues compared with adjacent normal tissues [109]. Overexpression of hsa_circ_0011290 in PTC compared with matched para-cancerous thyroid tissues was associated with advanced stage and poor prognosis of PTC [32]. Yao et al. demonstrated that overexpression of circ_0058124 in PTC tissues was associated with malignant features and poor outcomes in PTC patients in terms of advanced tumor-node-metastasis (TNM) stage, tumor size, LN metastasis, extrathyroidal extension, and distant metastasis [110]. Upregulation of circ_0005273 was reported in PTC tissues and was related to poor survival [64]. Overexpression of circ_BACH2 was reported in PTC tissues compared with adjacent tissues and was closely linked to larger tumor size, advanced TNM stage, lymph node metastasis, and shorter survival times [3]. Upregulated circ_0008274 was associated with poor PTC prognosis. Overexpression was associated with advanced TNM stage, tumor infiltration, and lymph node metastasis [111,112].

Similarly, circ_0067934 overexpression in TC tissues was associated with larger tumor size, higher AJCC stage, lymph node metastasis, and shorter survival times [113]. Wang et al. reported a lower level of circRNA itchy E3 ubiquitin-protein ligase (circ-ITCH) expressed in PTC tissues than in normal adjacent tissues [71]. In addition, circ_0137287 was downregulated in PTC tissues and related to aggressive clinical pathologic features in PTC patients, such as larger tumor size, advanced T stage, lymph node metastasis, and extrathyroidal extension [114] (Table 2).

**Table 2:** Role of circRNAs as diagnostic and prognostic biomarkers in PTC patients.

circRNAs	T	N	M	ETE	Clinical Stage	Poor Survival	Ref.
Upregulated biomarkers							
circ_PSD3							[109]
circ-UMAD1		●					[115]
circ_0058124	●	●	●	●	●		[110]
circ-NRIP1					●		[65]
circ_0005273						●	[64]
circ_0067934	●	●			●	●	[113]
circ_BACH2	●	●			●	●	[3]
circ_0008274		●			●		[111,112]
circ_0004458	●	●	●				[116]
circ_0062389	●	●					[117]
circ_0011290					●		[32]
circ-NRIP1					●		[65]
circ_0001666		●					[118]
Downregulated biomarkers							
circ_ITCH							[71]
circ_0137287	●	●		●			[114]

### 3.2. Circular RNAs Mediate Tumor Progression In Vivo and In Vitro

**Figure 4:**
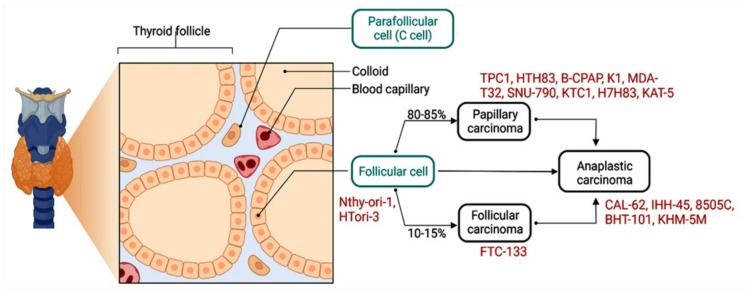
In vitro cell lines were used to study circRNA expression [126,127,128,129].

Emerging evidence suggests that some circRNAs play a significant role in tumor progression via multiple mechanisms, such as cell proliferation, differentiation, autophagy [119,120], EMT [56,78,121,122], immune modulation [51], apoptosis [114], vascularization [123], invasion, and metastasis [120,124]. The circRNAs–miRNA–mRNA axis can deregulate various cancer-related signaling pathways [29,125] (Figure 4).

#### 3.2.1. Tumor-Suppression-Related circRNAs

Some circRNAs were found to exert an anti-tumor function in PTC. In a microarray experiment, downregulated circRNA_100395 was observed in six PTC tumors compared with matched contralateral normal tissues. Circ_100395 showed interactive potential with two cancer-related miRNAs (miR-141-3p and miR-200a-3p), suggesting a role for the “hsa_circRNA_100395/miR-141-3p/miR-200a-3p” axis in PTC pathogenesis [130]. Moreover, downregulation of circ-ITCH was reported in “K1, IHH-4, and TCP-1” PTC cell lines [71]. Circ-ITCH promoted apoptosis in vitro and inhibited cancer cell proliferation in vivo, based on gain-of-functional assays. Circ-ITCH upregulates the expression of “casitas B-lineage lymphoma (CBL)”, an E3 ligase of nuclear β-catenin, by sponging miR-22-3p. Elevated levels of CBL suppressed the Wnt/β-catenin pathway activity with subsequent inhibition of PTC progression [71]. Another study analyzed microarray data from 18 thyroid samples and identified downregulated circRNA_100395 as a promising biomarker for papillary thyroid carcinoma through modulation of “miR-141-3p” and “miR-200a-3p” [130].

#### 3.2.2. Tumorigenesis-Related circRNAs

Other upregulated circRNAs are known to promote tumor growth (Table 3). CircTP53 is upregulated in TC cell lines, and this overexpression is associated with increased cancer cell proliferation and viability of TPC-1 cells. CircTP53 sponge miR-1233-3p thus increased “mouse double minute 2 (MDM2)” expression and downregulates the protein level of p53. Knockdown of circTP53 inhibited the expression of MDM2 and increased the protein level of p53 [30]. Jin and colleagues reported that circ_0004458 was overexpressed in PTC tissues and was associated with tumor size, invasion, lymphatic infiltration, and distal metastasis. Furthermore, expression levels were upregulated in different TC cells (BCPAP, TPC-1, K1, and IHH4) compared with NORI cells. In contrast, circ_0004458 silenced suppressed cell growth and enhanced apoptosis and cell cycle arrest in PTC cell lines in vitro, and hampered tumor growth in nude mice by inhibiting miR-885-5p and activating “Rac family small GTPase 1 (RAC1)” [116].

Han et al. reported circ_ABCB10 upregulation in PTC tissues relative to adjacent tissues. Circ_ABCB10 overexpression induced the proliferation/invasion of TC cells by targeting KLF6. The silencing of this circRNA promotes apoptosis both in vitro and in vivo and, therefore, can be considered for future targeted therapy in PTC [47]. Similarly, circ_0025033 was elevated in PTC. Pan et al. confirmed the role of circ_0025033 overexpression in promoting cell growth, invasion, and metastasis. Circ_0025033 sponges miR-1231 and miR-1304 to stimulate clone formation and inhibit apoptosis [48]. In another study, circ_0039411 was elevated in PTC and cell lines (K1, TPC-1, FTC-133, SW579). At the same time, circ_0039411 knockdown inhibited cell proliferation/motility and accelerated apoptosis in PTC cells by regulating miR-1179 and miR-1205, which regulate the expression of the lipid “ATP-binding cassette transporter A9 (ABCA9)” and “metastasis-associated 1 (MTA1)” [49].

Yao and colleagues identified overexpression of circ_0058124 in PTC tissues and cell lines (K1, TPC-1). Circ_0058124 can modulate miR-218-5p and *NUMB* (i.e., target gene) expression, which in turn regulates the “NOTCH3/GATAD2A” signaling axis both in vitro and in vivo, suggesting that circ_0058124 may be considered as a promising target for therapeutic intervening in the progression of PTC [110]. The circRNA BTB domain and CNC homolog 2 (circ-BACH2) were overexpressed in PTC cell lines relative to normal thyroid follicular epithelial cells (Nthyori 3–1). Circ-BACH2 was predominately localized in the cytoplasm and may sponge miR-139-5p with subsequent “LIM domain only 4 (LMO4)” upregulation and PTC development [3]. In another study by Liu and colleagues, the circ_0058124 was upregulated in PTC tissues and cells (IHH-4 and TPC-1), acting as an oncogene by mediating the “miR-370-3p/*LMO4*” axis. Its knockdown inhibited cell viability, colony formation, migration/invasion and blocked tumor growth in vivo, and promoted apoptosis in vitro [131].

**Table 3:** Upregulated circRNAs promote thyroid tumor progression.

CircRNA	Parental Gene	Model	Source	miRNA	Target Gene	Mechanism	Ref.
circ-TP53	*TP53*	In vitro, in vivo	TPC-1	↓miR-1233-3p	↑*MDM2*, ↓*TP53*	Promotes cancer cell viability and proliferation	[30]
circ_0004458		In vitro, in vivo, human (N = 48)	BCPAP, TPC-1, K1, IHH4	↓miR-885-5p	↑*RAC1*	Promotes cell growth and inhibits apoptosis and cell cycle arrest	[116]
circ-ABCB10	*ABCB10*	In vitro, in vivo, human (N = 40)			↓*KLF6*	Enhances proliferation and invasion	[47]
circ_0025033		In vitro		↓miR-1233, miR-1304		Promotes cell growth, invasion, and metastasis, triggers clone formation and prevents apoptosis	[48]
circ_0039411		In vitro, human (N = 46)	K1, TPC-1, FTC-133, SW579	↓miR-1179, miR-1205	↑ *MTA1*, *ABCA9*	Promotes cell proliferation/motility and inhibits apoptosis	[49]
circ-BACH2	*BACH2*	In vitro		↓miR-139-5p	↑*LMO4*	Initiates tumor	[3]
circ_0058124		In vivo, in vitro, human (N = 92)	TPC-1, K1	↓miR-218-5p	↑*NUMB*, *NOTCH3*, *GATAD2A*	Acts as oncogene	[110]
circ_0058124		In vitro, in vivo	TPC-1, IHH-4	↓miR-370-3p	↑*LMO4*	Promotes progression, colony formation, migration, invasion, inhibits apoptosis and tumor growth	[131]
circ_0058124		In vitro, in vivo, human (N = 51)	BCPAP, TPC-1, IHH-4, HTH83	↓miR-940		Acts as oncogene	[28]
circ-LDLR	*LDLR*	In vitro, in vivo, human (N = 60)		↓miR-195-5p	↑*LIPH*	Promotes malignant behavior, colony formation, proliferation, migration, and invasion, and inhibits apoptosis	[132]
circ_ZFR	*ZFR*	In vitro, human (N = 41)	k1, TPC-1, SW579, FTC133	↓miR-1261	↑*C8orf4*	Triggers tumor growth	[31]
circ_102171		In vitro, in vivo, human (N = 47)	TPC-1, NPA87, KAT-5		↓*CTNNB1P1*, *TCF3*, *TCF4*, *LEF1*	Promotes progression, cell proliferation, migration, activation of the beta-catenin pathway, inhibits apoptosis	[27]
circ_0011290		In vitro	NIM-1, HTH83, TPC-1, K1, BCPAP	↓miR-1252	↑*FSTL1*	Promotes cell viability, proliferation, apoptosis, and glucose metabolisms	[32]
circ_0058129		In vitro, in vivo, human (N = 70)	TPC-1, SNU-790	↓miR-873-5p	↑*FSTL1*	Fosters cell proliferation, invasion, and migration	[133]
circ_0005273		In vitro	KTC-1, IHH-4, BCPAP, TPC-1	↓miR-1138	↑*SOX2*	Promotes cell viability, proliferation, invasion, and migration	[64]
circ_0005273		In vitro, human		↓miR-1138		Acts as oncogene	[113]
circ-NEK6	*NEK6*			↓miR-370-3p	↑*FZD8*	Promotes progression, invasion, and metastasis, activates Wnt signaling pathway	[73]
circ-FAT1(e2)	*FATI*			↓miR-873	↑*ZEB1*	Promotes PTC cell growth, migration, and invasion	[62]
circ-PSD3	*PSD3*	In vitro, in vivo		↓miR-637	↑ *HEMGN*	Activates PI3K/Akt signaling, promote cell cycle progression, proliferation and metastasis and impedes apoptosis	[109]
circ_0008274		In vitro, in vivo		↓miR-154-3p	↑*SLC7A11*	Regulates apoptosis, migration, and adhesion	[111]
circ_0008274		In vitro, human			↑*AMPK*, *mTOR*	Promotes proliferation/invasion through the “AMPK/mTOR” signaling pathway	[112]
circ_0067934		In vitro, human (N = 57)			↑*PI3K*, *AKT*	Promotes EMT, PI3K/AKT signaling pathway, cell proliferation, invasion, migration, and inhibits apoptosis.	[121]
circ_0067934		In vitro, in vivo		↓miR-1304	*CXCR1*, *PRKCI*	Increases cell proliferation, migration, and invasion	[113]
circ_0067934							
circ-DOCK1	*DOCK1*	In vitro	FTC-133, TPC-1	↓miR-124		Induces tumorigenesis	[134]
circ-PRMT5	*PRMT5*	In vitro	K1, TPC-1, IHH4, BCPAP cells	↓miR-30c		Induces tumorigenesis	[135]
circ-FOXM1	*FOXM1*	In vitro, in vivo, human (N = 78)	K1, IHH-4, BCPAP, TCP-1	↓miR-1179		Increases tumor growth	[136]
circ_0011385		In vitro, in vivo	BCPAP	↓miR-361-3p		Increases cell proliferation, invasion, and migration, inhibits apoptosis and regulates metastasis-related proteins	[137]
circ-RAPGEF5	*RAPGEF5*	In vitro, in vivo	BCPAP, KTC-1, K1, HEK293	↓miR-198	↑*FGFR1*	Knockdown inhibits cell proliferation, migration, and invasion	[138]
circ_0103552		In vitro, human (N = 56)	TPC-1, SW579, 8505C			Regulator for invasion and migration	[139]
circ-UMAD1	*UMAD1*	Human (N = 50)		↓miR-873	↑*Gal3*	Poor prognosis, lymph node metastasis	[115]
circ_IPCEF1		Human (N = 50)		↓miR-3619-5p	↑*CASR*, *CDC25B*, *NFκB1* and *SHOC2*	Of 158 deregulated circRNAs, it showed the best predictive power	[140]
circ_0001666		In vitro, in vivo, human		↓miR-330-5p, miR-193a-5p, miR-326	↑*ETV4*	Enhances G_1_ phase cell cycle progression	[118]
circ-UBAP2	*UBAP2*	In vitro		↓miR-370-3p		Regulates proliferation, apoptosis, and invasion	[141]
circ-PVT1	*PVT1*	In vitro		↓ miR-195	↑*VEGFA*	Activates the Wnt/β-catenin signaling pathway	[142]
circ-PUM1	*PUM1*	In vitro		↓miR-21	↑*MAPK1*	Promotes cell growth, metastasis, and glycolysis	[143]
circ-PRKCI	*PRKCI*			↓miR-335	↑*E2F3*, *MMP2*, *MMP9*, *snail*	Promotes cell progression and glycolysis	[66]
circ_0079558		In vitro, human		↓miR-26b-5p	↑*MET*/*AKT*	Facilitates the proliferation and motility	[144]
circ-RPS28	*RPS28*	In vitro		↓miR-345-5p	↑*FZD8*, *MMP2*, *MMP9*, *BAX1*	Regulates cell growth and motility	[145]
circ-NRIP1	*NRIP1*	In vitro, in vivo, human		↓miR-195-5p	↑*p38*, *JAK2*, *STAT1*	Promotes cell proliferation/invasion and inhibits apoptosis	[65]
circ_0001018		In vitro		↓ miR-338-3p	↑*SOX4*	Facilitates cell survival, invasion, G1/S cell cycle progression, and represses cell apoptosis	[63]
circ_0067934		In vitro, in vivo, human		↓ miR-1301-3p	↑*HMGB1*	Increases growth, colony formation, migration, invasion, EMT	[146]
circ_0062389		In vitro, human		↓miR-1179	↑*HMGB1*	Promotes proliferation, migration, and EMT	[117]
circ-HIPK3		In vitro		↓miR-338-3p	↑*RAB23*	Promotes invasiveness, migration, invasion, and proliferation	[147]
circ_0059354		In vitro, in vivo		↓miR-766-3p	↑*ARFGEF1*	Aggravates cell proliferation, migration, invasion, and angiogenesis	[123]
circ-NEURL4	*NEURL4*			↓ miR-1278	↑*LATS1*	Enhances cell proliferation and invasion	[148]
circ-TIAM1	*TIAM1*	In vitro, in vivo, human (N = 60)	K1, TPC-1, IHH-4, B-CPAP	↓miR-646	↑*hnRNPA1*	Promotes cell migration, adhesion, growth, and polarity	[149]
circ-ITGA7	*ITGA7*	In vitro	TPC1 and CAL-62	↓miR-198	↑*FGFR1*	Promotes migration and invasion	[72]
ciRS-7				↓miR-7	↑*EGFR*	Promotes tumor growth, EMT	[78]
circ_0001681				↓miR-942-5p	↑*TWIST1*		[150]

Circ-LDLR is overexpressed in PTC tissues/cells. This overexpression regulated Lipase H (LIPH) expression by sponging miR-195-5p. Knockdown of this circRNA suppressed PTC cells’ malignant behaviors and promoted PTC cell colony apoptosis in vivo [132]. Circ_ZFR was also implicated in thyroid cancer. It can sponge and silence miR-1261, allowing the activation of the transcriptional and immune response regulator C8orf4 and triggering cancer cell growth [31]. The role of circ_102171 in PTC tissues and cell lines (TPC-1, NPA87, KAT-5) has also been examined. Higher expression was shown in PTC samples. Circ_102171 accelerated PTC progression by modulating “CTNNB1P1-dependent beta-catenin” pathway activation. It can target CTNNBIP1 by suppressing the “β-catenin/TCF3/TCF4/LEF1” pathway to initiate the “Wnt/β-catenin” pathway and progression of PTC. CircRNA_102171 downregulation decreased cell proliferation/migration and triggered apoptosis in vivo and in vitro [27].

Overexpression of circ_0011290 was reported in PTC cell lines (NIM-1, HTH83, TPC-1, K1, BCPAP). In vitro, circ_0011290 could positively modulate the protease inhibitor follistatin-like 1 (FSTL1) expression through sponging miR-1252. Suppression of miR-1252 reversed the phenotypic impacts of circ_0011290 silencing, including cell viability/proliferation and glucose metabolism [32]. Similarly, in PTC tissues and cells, circ_0058129 and FSTL1 abundances were increased, whereas the miR-873-5p content was decreased relative to control groups. Circ_0058129 shortage suppressed PTC cell proliferation/invasion and migration. Moreover, miR-873-5p repressed PTC cell malignancy by binding to FSTL1. Circ_0058129 targeted miR-873-5p to regulate FSTL1 [133]. Circ_0005273 was overexpressed in PTC cell lines (KTC-1, IHH-4, BCPAP, TPC-1). It can bind to miR-1138 and relieve suppression of “SRY (sex-determining region Y)-box 2 (SOX2)”. Knockdown of circ_0005273 hindered cell viability, proliferation, invasion, and migration of PTC. Silencing of circ_0005273 could suppress PTC tumor growth in vivo, making this circRNA a potential new therapeutic target for PTC [64,113].

By targeting miR-370-3p, Circ-NEK6 promotes cancer cell invasion, metastasis, and disease progression via transmembrane signaling receptor “frizzled family receptor 8 (FZD8)” upregulation and “Wnt signaling” pathway activation in TC [73]. CircFAT1(e2), derived from exon 2 of the *FAT1* gene, was upregulated in PTC cell lines, and its knockdown inhibited PTC cell growth/invasion and migration. CircFAT1(e2) can sponge miR-873, promoting *ZEB1* expression, which modulates cancer progression, invasion, and metastasis [62]. Circ_PSD3 was upregulated in PTC cell lines (TPC-1, CGTH-W3, IHH-4, SW579). Circ_PSD3 enhances PTC progression by regulating the “miR-637/HEMGN” axis and activating “PI3K/Akt” signaling. Circ_PSD3 and HEMGN facilitate cell cycle progression, proliferation, and metastasis and impedes PTC cell apoptosis. Circ_PSD3 silencing hampered tumor growth in vivo and facilitated apoptosis in vitro [109].

Zhou et al. suggested circ_0008274 to be a putative tumor-promoting circRNA in PTC. It was found to regulate PTC cell migration/adhesion and apoptosis in vitro. Its knockdown inhibited PTC cell migration/adhesion and enhanced apoptosis in vitro, and restrained tumor growth in vivo. At the cellular level, circ_0008274 modulated “solute carrier family 7-member A11 (SLC7A11)” expression by acting as a sponge of miR-154-3p, releasing the inhibiting effect on the transmembrane transporter SLC7A11. Hence, regulating the “miR-154-3p/SLC7A11” axis might provide a promising therapeutic target for PTC treatment [111]. Similar results were obtained by Zhou et al.; circ_0008274 was also significantly higher in PTC tissues and cell lines and was associated with poor prognosis. Circ_0008274 enhances PTC cell proliferation/invasion by regulating the “AMPK/mTOR” signaling pathway [112].

Circ_0067934 was highly expressed in TC cell lines. It acts as an oncogene, leading to the development of TC by promoting the EMT and “phosphatidylinositol 3-kinase (PI3K)/protein kinase B (AKT)” signaling pathways [113]. Knockdown of circ_0067934 inhibited cell proliferation/migration and invasion and enhanced apoptosis [121]. By analogy to cell lines, circ_0067934 RNA was also highly expressed in TC compared with adjacent non-cancer tissues. Its overexpression was associated with shorter patient survival. Silencing of this circRNA inhibited cell proliferation and triggered apoptosis, as evidenced by cell counting/Transwell migration assays. Western blot results suggested this knockdown also prevented EMT and PI3K/AKT-pathway-related protein synthesis [113,121].

Circ_0011385 is overexpressed in TC. Its silencing inhibited TC cell proliferation/migration and invasion and supported cell cycle arrest/apoptosis. It has been reported to act as a sponge for miR-361-3p with negative regulation in vitro. It was also implicated in regulating several apoptosis/metastasis-related proteins in TC [137], supporting the value of the “circ_0011385/miR-361-3p” axis as a novel therapeutic opportunity for TC.

Circ-RAPGEF5 is a transcript of a five-exon *RAPGEF5* gene expressed in PTC cells with uncertain oncogenic properties. Liu et al. evaluated the potential participation of this circRNA in TC pathogenesis and explored its upregulation in PTC tissues and cultures. They claimed that *RAPGEF5* suppression could attenuate the transmembrane signaling receptor “fibroblast growth factor receptor 1 (FGFR1)” and thus suppress tumor growth by binding to miR-198, which sponged the 3′-UTR of FGFR1 via circRAPGEF5 targets in vitro. Xenograft experiments suggest that silencing circRAPGEF5 by prompting miR-198 expression can inhibit the oncogenic potential of FGFR1, making the “circRAPGEF5/miR-198/FGFR1” axis a promising target for PTC treatment [138]. Further studies showed that circ-0103552 was involved in the invasion/migration of TC via miR-127 sponging [139].

The concomitant presence of circulating circRNA-UMAD1 and Galectin-3 is considered a “co-biomarker” for predicting TC lymph node metastasis [115]. CircRNA profiling by microarray revealed a potential role of circ_IPCEF1 in PTC and showed interactions with four cancer-related genes, “*CASR*, *CDC25B*, *NFκB1*, and *SHOC2*” through sponging miR-3619-5p [140]. Additionally, circ_0001666 was upregulated in both PTC tissues and cell lines, and its expression was associated with lymph node metastasis. Silencing circ_0001666 inhibited cell proliferation, increased cell-cycle-associated proteins, arrested cell cycle progression at the G1 phase, and enhanced the expression of proapoptotic proteins. The oncogenic effect of circ_0001666 on tumor growth was also verified in xenograft experiments [118].

In another study, circUBAP2 was upregulated in TC tissues/cells, whereas its target miR-370-3p was downregulated. Silencing of this circRNA suppressed cell proliferation/invasion and induced apoptosis in TC cells [141]. Circular RNA Pvt1 oncogene (CircPVT1) supports the PTC progression by triggering the “Wnt/β-catenin” signaling pathway and modulating the ratio of miR-195/VEGFA expression [142]. Silencing of circPUM1 impairs cell growth, tissue glycolysis, and metastasis of PTC by sponging of miR-21-5p ensuing MAPK1 upregulation [143].

Knockdown of circRNA protein kinase C iota (circ-PRKCI) inhibits cell progression/glycolysis of PTC via the “circ-PRKCI/miR-335/E2F3” ceRNA axis [66]. Xenograft experiments indicated that the knockdown of this circRNA could hinder PTC cell proliferation and tumor growth in vivo [66]. Circ_0079558 stimulates PTC progression and facilitated proliferation/motility by sponging miR-26b-5p with activation of MET/AKT signaling [144]. CircRPS28 (hsa_circ_0049055) is an emerging player in regulating cell growth/motility of PTC by sponging miR-345-5p to regulate FZD8 [145]. Silencing this type of circRNA retained PTC cell viability, wound-healing rate, and numbers of colony formation and migration/invasion cells associated with apoptosis promotion. These effects are in line with decreased “B-cell lymphoma (Bcl)-2” levels and increased “Bcl-2-associated X protein (Bax), matrix metalloproteinase-2 (MMP2), and MMP9” levels as explored by Western blotting [145].

CircRNA NRIP1 was upregulated in PTC tissues/cells, and its high levels were associated with advanced PTC stage. Silencing of this circRNA suppressed PTC cell proliferation/invasion while accelerating apoptosis. Overexpression of miR-195-5p inhibited proliferation/invasion capabilities, triggering apoptosis of PTC cell lines, and retained the growth of tumor xenografts. These functions were reversed following circRNA NRIP1 upregulation in PTC cells/tumor xenografts. Protein levels of p38/JAK2/STAT1 were markedly downregulated in miR-195-5p overexpressed PTC cells/tumor xenografts, whereas circRNA NRIP1 overexpression negated these impacts [65].

Circ_0001018 promotes PTC by promoting cell survival/invasion, G1/S cell cycle progression, and inhibiting cell apoptosis via crosstalk with miR-338-3p and SOX4 [63]. Circ_0067934 supports the PTC cells’ progression via the “miR-1301-3p/high mobility group box 1 (HMGB1)” axis. Silencing of circ_0067934 inhibited growth, colony formation, migration/invasion, EMT, and growth of tumor xenograft and triggered apoptosis of PTC cells [146]. Circ_0062389 regulates PTC progression via the “miR-1179/HMGB1” axis and is significantly overexpressed in PTC tissues/cell lines. Upregulation of this circRNA was associated with large tumor size and positive lymph node metastasis. Silencing circ_0062389 could impede the PTC cells’ proliferation/migration and EMT [117].

In TC, another study suggested the potential role of circ_0001681 in the EMT process. This cytoplasmic circRNA was upregulated in TC cells and inversely related to the miR-942-5p level [150]. By targeting miR-942-5p and upregulating the TWIST1 expression, silencing of circ_0001681 significantly suppressed TC progression [150]. These findings indicate that circ_0001681 is a promising treatment candidate for TC.

The oncogenic circ_0059354 has been implicated in PTC progression by upregulating ARFGEF1 through miR-766-3p sponging. Circ_0059354 silencing facilitated apoptosis and suppressed cell proliferation/migration, invasion, and angiogenesis in PTC cells [123]. Inhibition of miR-766-3p reversed circ_0059354-knockdown-mediated effects, whereas its overexpression restrained the PTC cells’ malignant behaviors. At the same time, downregulation of circ_0059354 blocked tumor growth in vivo [123].

Circ-NRIP1 promotes the progress of PTC via sponging mir-195-5p and modulating the “P38 MAPK and JAK/STAT” pathways [65]. This circRNA was overexpressed in PTC tissues/cells, and its levels correlated with advanced stages of PTC. Silencing of this circRNA suppresses PTC cell proliferation/invasion while accelerating apoptosis. Upregulation of miR-195-5p repressed proliferation/invasion capabilities, accelerated apoptosis of PTC cell lines, and restrained the growth of tumor xenografts. In addition, protein levels of “p-p38, p-JAK2, and p-STAT1” were downregulated in miR-195-5p-overexpressed PTC cells and tumor xenografts, whereas CircRNA NRIP1 upregulation reversed these effects [65]. Furthermore, circHIPK3 sponges miR-338-3p and enhance RAB23 expression in TC cells, promoting cancer invasiveness. Silencing of circHIPK3 significantly reduced proliferation/invasion and migration of TC cells [147].

Circ_0079558 and MET were reported to be upregulated in PTC tissues/cell lines. This circRNA promoted PTC cell proliferation/migration and decreased apoptosis through the “miR-26b-5p/MET/AKT” axis [144]. Circ-TIAM1 induced cell migration of PTC via the “miR-646/heterogeneous ribonucleoprotein A1 (HNRNPA1)” axis [149]. Meanwhile, circNEURL4 downregulation in the PTC samples was directly associated with an unfavorable prognosis. Through binding to miR-1278, circNEURL4 might suppress cell proliferation/invasion of PTC in vivo and in vitro by indirectly increasing the expression of LATS1 [148]. Thus, this circRNA could have a clinical diagnostic and/or prognostic utility for PTC patients and a potential targeted therapeutic for PTC via the “miR-1278/LATS1” axis.

### 3.3. Tumor-Cell-Derived Exosomal circRNAs

Exosomes are created in multivesicular endosomes and can be excreted from several cellular types. They are implicated in intercellular communication by transferring intracellular cargoes, such as proteins/nucleic acids [151]. RNA-seq analyses indicated that circRNAs were enriched in the exosomal compartment compared with the original cells [44,45], and a further study has identified that circRNA levels in exosomes could mirror their levels in cells/tissues [57]. Also, circRNAs sorting to exosomes may be controlled by changes in the levels of associated miRNA in producer cells, and the biological activities could be transferred to recipient cells. The abundance of tumor-derived serum “exosomal circRNAs (exo-circRNA)” in xenografted mice was correlated with tumor mass [44]. Exo-circRNAs could discriminate patients with colon cancer from healthy controls, suggesting the putative biological function of exosomal circRNAs [92]. Emerging evidence shows the excellent promise of exosomal circRNAs in tumor immunity regulation, patient outcome prediction, and drug efficacy evaluation [152].

Exosomal circ_007293 was reported to promote proliferation/invasion, migration, and EMT of PTC cells through regulation of the “miR-653-5p/paired box 6 (PAX6)” axis [153], suggesting that tumor-derived exosomal circRNAs may function as potential biomarkers for cancer progression. Additionally, “circRNA-UMAD1 and Gal3” were recognized as having more substantial co-biomarker potential with significant upregulation in the sera of patients with LNM, relative to those with primary tumors, as verified by the serum RNA expression levels of 50 patients with PTC associated with or without LNM by “quantitative real-time PCR” [115]. Further studies using liquid biopsy samples are recommended to facilitate dynamic monitoring of cancer and postoperative surveillance.

### 3.4. Circular RNAs and Treatment Resistance

Since the sensitivity of tumors to chemotherapy can impact the survival/prognosis of patients, deciphering the mechanisms underlying drug resistance is critical. Dysregulation of circRNAs in cancer was found to induce chemoresistance. CircHIPK3 overexpression was found to promote gemcitabine (GEM) resistance in pancreatic cancer cells by targeting “RASSF1” through miR-330-5p and was proposed to be a novel biomarker in GEM-resistant PC [154]. Similarly, “circRNA Cdr1as” induced apoptosis and increased the cisplatin chemosensitivity of bladder cancer cells both in vitro and in vivo by upregulating “APAF1” expression through miR-1270 inhibition [155]. Sang et al. indicated that circRNA_0025202 modulated tamoxifen sensitivity and tumor progress through the “miR-182-5p/FOXO3a” signaling pathway in breast cancer [156].

**Figure 5:**
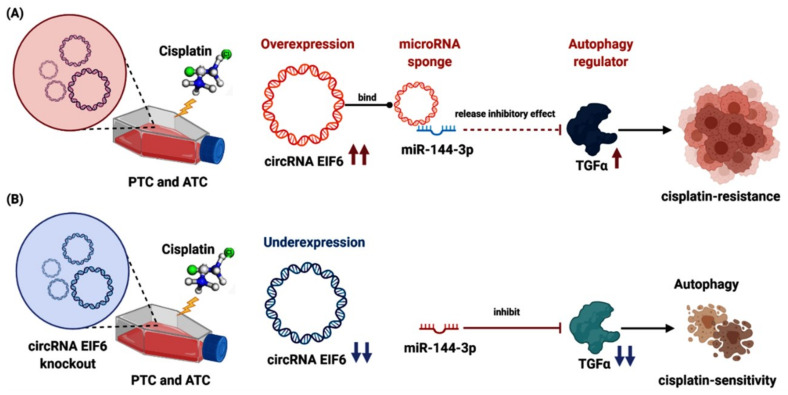
CircRNAs and chemotherapy drugs in thyroid cancer. (**A**) The role of overexpressed “circEIF6”, its target miR-144-3p, and downstream “transforming growth factor-α (TGF-α)” in cisplatin resistance in papillary and anaplastic thyroid cancer cell lines. CircEIF6 sponges miR-144-3p to promote the cisplatin resistance of human TC cells by autophagy regulation. TGF-α activation accelerates the carcinogenesis process and cisplatin resistance. (**B**) Knockout of “circEIF6” reverses the processes mentioned above, sensitizing the papillary and anaplastic thyroid cancer cell lines to cisplatin (Created by Biorender.com).

In thyroid carcinoma, Liu et al. [119] recognized that “circRNA EIF6 (hsa_circ_0060060)” sponges miR-144-3p to promote resistance to cisplatin in PTC and ATC cell lines by regulation of autophagy. CircEIF6 targets hsa-miR-144-3p, which modulates the expression of transforming growth factor α (TGF-α) and other autophagy-related proteins. In contrast, circEIF6 silencing augmented cisplatin sensitivity in vivo by mediating autophagy [119]. The above studies indicate that circRNAs are promising therapeutic targets for reversing drug resistance in tumors (Figure 5).

### 3.5. Functional Enrichment Analysis for the Deregulated Markers

**Figure 6:**
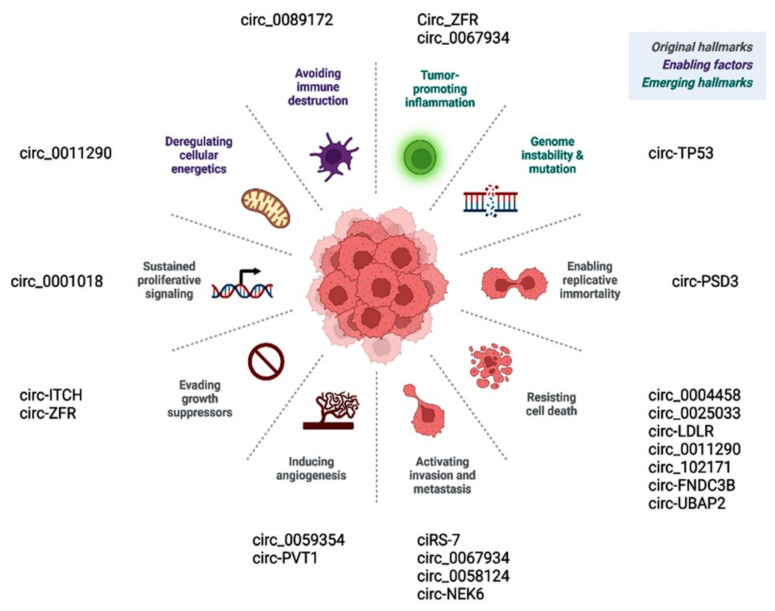
CircRNAs regulate various hallmarks of cancer.

Prior publications have shown the role of deregulated circular RNAs in various hallmarks of cancer (Figure 6). Circ_0001018 activates cell proliferation by regulating the miR-338-3p/SOX4 axis [63]. Circ-ITCH and similar circRNAs promote cancer cells, evading antigrowth signals by enhancing other oncogenes, such as CBL [71]. CircEIF6/miR-144-3p promotes cancer cells by evading cell death via regulating cellular apoptosis or autophagy [119]. Circ-PSD3 limits the replicative potential of cancer cells and impedes apoptosis by regulating HEMGN [109]. Circ_0059354/miR-766-3p sustains angiogenesis through regulating ARFGEF1 [123]. CiRS-7/miR-7/EGFR [78], circ_0067934/PI3K/AKT [121], circ_0067934/miR-1301-3p/HMGB1 [146], and circ_0062389/miR-1179/HMGB1 [117] regulate the EMT process and thus cancer tissue invasion/metastasis.

**Figure 7:**
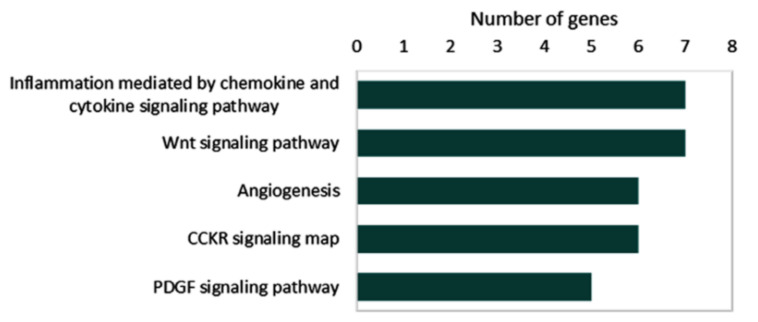
Pathway enrichment analysis for overexpressed genes caused by upregulated circRNAs. Data source: “PANTHER (Protein ANalysis THrough Evolutionary Relationships) Classification System (http://pantherdb.org (accessed on 23 June 2022))”.

Collectively, 34 microRNAs and 51 target genes are known to be deregulated by circRNAs in thyroid cancer. Pathway enrichment analysis of 51 target genes in the reviewed articles demonstrated their role in inflammation mediated by chemokine and cytokine signaling (MAPK1, JAK2, TCF3, STAT1, RAC1, CXCR1, and p38), the Wnt signaling pathway (TCF3, PRKC1, TP53, FSTL1, FZD8, and LEF1), angiogenesis (MAPK1, FGFR1, VEGFA, p38, STAT1, and PRKCI), CCKR signaling map (MAPK1, JAK1, TCF4, MP9, p38, and RAC1), and the PDGF signaling pathway (MAPK1, JAK2, mTOR < STAT1, and p38) (Figure 7).

### 3.6. Genome-Wide Circular RNA Screening

#### 3.6.1. Data Source and Processing

Microarray transcriptomic signature of circRNAs in human PTC was obtained from the “Gene Expression Omnibus database (http://www.ncbi.nlm.nih.gov/geo/)”. A systematic search revealed a single dataset for circRNA analysis in TC (GSE93522). It contains 18 samples, of which 6 PTCs were compared with their matching contralateral normal thyroid tissues. The platform used was “GPL19978 Agilent-069978 Arraystar Human CircRNA microarray V1”.

The raw data were analyzed via GEO2R software (http://www.ncbi.nlm.nih.gov/geo/geo2r/) using R version 3.2.3 software packages (Biobase 2.30.0, GEOquery 2.40.0, limma 3.26.8), and the results were confirmed using NetworkAnalyst (www.networkanalyst.ca). Differentially expressed circRNA (DEC) analysis was performed using a *p*-value threshold of <0.05 and log fold change (FC) >1. Annotation files from the platform were used to identify the corresponding circRNA names of the probes, and genes from which circRNAs are derived were identified using circBase (www.circBase.org). A volcano plot was used to visualize the significantly differential circRNAs between PTC and controls. A heatmap was constructed to display the differential circRNA expression patterns among the samples under R version 4.0.3 using the matrix data. DEC was plotted on ideograms using Phenograms (https://ritchielab.org/software/phenogram) (all online tools were last accessed on 23 June 2022).

#### 3.6.2. Identification of Differentially Expressed circRNAs (DEC)

**Figure 8:**
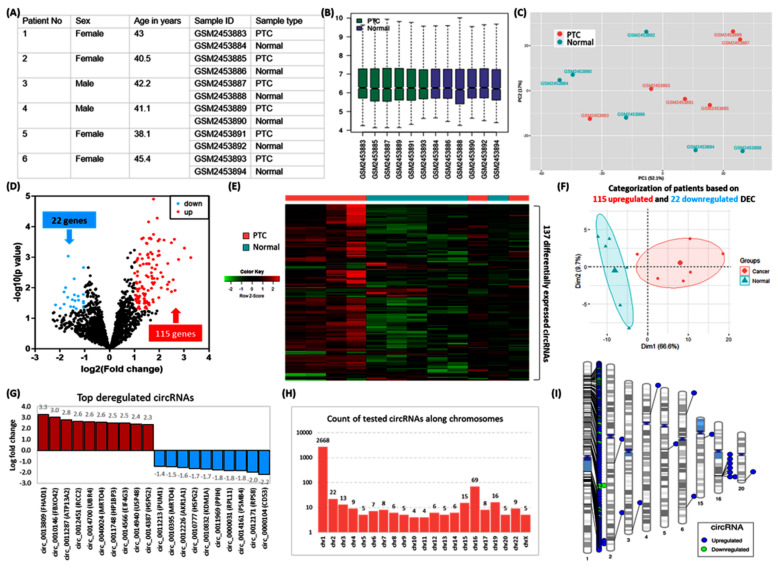
Microarray transcriptomic signature of circRNAs in human papillary thyroid carcinoma. (**A**) Characteristics of six PTC patients were retrieved from the Gene Expression Omnibus database (GSE93522). PTC was compared with their paired contralateral normal thyroid tissues (**B**) Boxplots showing the distribution of the values of the selected samples. Median-centered values are indicated after log transform and quantile normalization. (**C**) Principal component analysis for data exploration. The geometrical projection for samples is based on the similarities and differences in the expression of 2895 circRNAs. Samples are plotted across two coordinates; axis 1 explained 52.1% of the variance, whereas axis 2 demonstrated 17% of the data variability. (**D**) Volcano plot displays statistical significance (−log10 *p*-value) versus magnitude of change (log2 fold change) in PTC compared with controls. Each dot represents a circRNA. The plot shows 137 significantly differentially expressed circRNAs (DEC): 115 upregulated (red dots) and 22 downregulated (blue dots). Analysis was performed using GEO2R software, with the *p*-value threshold at <0.05 and log fold changes (FC) at >1. (**E**) Heatmap for the 12 thyroid tissue specimens using the 137 differentially expressed circRNAs. There was an upregulation pattern of genes in patient samples. Clear differentiation of four PTC tissues and incomplete separation of two others are shown. (**F**) Principal component analysis to visualize how samples are related to each other. By using the deregulated genes, a complete demarcation was observed between PTC and normal tissues. Axes 1 and 2 explain 66.6% and 9.7% of the variability, respectively. (**G**) The top-up and downregulated circRNAs in PTC tissues. Derived protein-coding genes from which circRNAs were formed are shown. Fold change values are depicted at the end of the bars. (**H**) Frequency of tested circRNAs in the microarray according to their chromosomal localization. Y axis is log10 transformed, and the X axis represents the human chromosomes with removed missing chromosomes (chromosomes 18, 21, and Y). (**I**) Chromosomal ideograms represent the differentially expressed circRNAs annotated with lines in color at specific base-pair locations: blue for upregulated DECs and green for downregulated DECs.

Characteristics of human thyroid specimens are shown in Figure 8A–C. In the current analysis, we identified 137 significant DEC in PTC compared with controls (Figure 8D). The expression pattern of these DEC enables clear demarcation between cancer and normal thyroid tissues (Figure 8E–F). The top 10 downregulated circRNAs were circ_0000104, circ_0012171, circ_0014161, circ_0000031, circ_0011969, circ_0010832, circ_0010777, circ_0012226, circ_0010395, circ_0011213. The top upregulated genes were circ_0013809, circ_0010146, circ_0011287, circ_0012451, circ_0014700, circ_0040024, circ_0011748, circ_0014566, circ_0014940, circ_0014387 (Figure 8G). Their spliced mature sequence length ranged from 71 to 141,103 base pairs. A total of 74 are transcribed from the antisense strand, whereas 63 DEC are generated from the sense strand. Of 2,895 studied circRNAs, 92.2% (N = 2668) were located along chromosome 1, and 2.38% of genes existed in chromosome 16 (N = 69) (Figure 8H). These included 115 upregulated genes, of which 105 circRNAs lie on chromosome 1. In contrast, the 22 downregulated circRNAs are in chromosome 1. Significant circRNAs were clustered at the short arm of chromosome 1 (1p13.3, 1p32.2), 1q21.3, and 1p34.2 to 1p36.13), long arm of chromosome 1 (1q22), and chromosome 16q22.1. (Figure 8I). The list of DECs and their annotation is shown in Supplementary Table S1.

## 4. Discussion of the Current Challenges in Clinical Practice and Future Perspectives

The first wave of human disease circRNA research showed great potential in human diseases. Moving forward, a better functional and clinical understanding of circRNA requires the implementation of best practice principles in future research; however, some experimental challenges should be considered. For purification, standard RNA preparation kits can isolate circRNAs [20], but circRNA will be largely missing in poly(A)-enriched samples as they lack poly(A) tails.

On the other hand, the removal of linear RNA challenges the assessment of whether the linear host genome and the circRNA expression are independent of one another [20]. It may be beneficial to begin with total RNA but allow enrichment for circRNA using an RNAase R treatment with Li^+^ (rather than K^+^) [157], poly(A) counterselection [158], and rRNA removal to increase the number of circRNA reads [20]. Assessment of RNA quantity, quality, and purity requires different profiling methods for circRNA [159].

When quantifying circRNA in RNA-seq data, the overlay is preferred between more than one bioinformatic algorithm (such as find_circ, CIRI2, CIRCexplorer2, and circRNA_finder) to minimize the potential for false-positive circRNAs [160]. Additional validation measures to examine the circular structure should also be undertaken. When detecting circRNAs using a microscope, fixed cells should be treated with a protease prior to probe hybridization to remove their association with ribosome binding proteins (RBP), which may inhibit probe binding and result in the absence of signals. Positive and negative controls should be included to check probe specificity [20].

Since circRNA expression is often specific to cell type, developing techniques for single-cell profiling or spatial transcriptomics for circRNAs offers resolution at a single-cell level, which is especially important in complex tumor tissues. Validating and predicting gene and drug targets will provide comprehensive mechanistic approaches. Although detection methods are still in their infancy, primarily confined to microarray analysis, early generation sequencing analyses suggest the possibility of circRNA detection transcriptome wide. Currently, circRNA validation is based mainly on circular structure confirmation and detection of its back splice junction. Moving forward, more accurate circRNA detection and validation methods are an area of interest in this field.

One of the limitations we had in the current analysis was the lack of circRNA detection in patients with other histopathological variants such as anaplastic, follicular, or medullary thyroid cancers. Moreover, studies tracing dynamic longitudinal monitoring of circRNAs during surveillance, postoperatively, or following radiotherapy were unavailable. The discovery of other circRNAs and elucidating the factors mentioned above will significantly improve the future of gene-targeted therapy.

## 5. Conclusions

Cancer remains a significant challenge for healthcare providers. Advances in its diagnosis and treatment are critical moving forward. This review aimed to elucidate TC development, progression, and treatment concerning circRNAs. The literature has demonstrated the benefits of using circRNAs as stable and tissue-specific biomarkers. Although the field is still in its early stages, we believe that circRNAs could serve essential roles in the diagnosis, prognosis, and treatment of cancers.

This work summarized the role of circRNAs in thyroid cancer and their ability to act as miRNA sponges, regulate transcription and translation, and serve as protein decoys. Consequently, subsequent determination of circRNA deregulation mechanisms could optimize their utility in the diagnosis, prognosis, and treatment of thyroid cancer. Importantly, circRNAs can be detected in patient plasma and potentially be used to track cancer progression. The advantages of circRNA, including its higher stability, wider abundance, and occurrence in several body fluids, potentiate its use as promising prognostic and theranostic biomarkers in TC.

It has been established that circRNAs interact with miRNAs to influence downstream targets playing vital roles in TC, such as metastasis, angiogenesis, and therapeutic resistance. Future work to elucidate these regulatory networks, as well as the significance of their manipulation and exploitation, will provide a comprehensive “Rosetta Stone” of this RNA dialect.

## Supplementary Materials

The following supporting information can be downloaded at: https://www.mdpi.com/article/10.3390/cancers14194728/s1, Table S1: Fold change and annotation of differentially expressed circRNAs in PTC compared with normal thyroid tissues.
