# Supplementary material for: A Review and In Silico Analysis of Tissue and Exosomal Circular RNAs: Opportunities and Challenges in Thyroid Cancer
Source: Cancers (Basel). 2022 Sep 28;14(19):4728. doi: 10.3390/cancers14194728 (PMC9564022; doi:10.3390/cancers14194728)
Supplement: Supplementary file 1 [file cancers-14-04728-s001.zip › cancers-1815416-supplementary.pdf]

Table S1. Fold change and annotation of differentially expressed cirRNAs in PTC compared to normal thyroid tissues

circID	logFC	*P*	Derived gene	chr	start	end	strand	genomic length	spliced seq length
hsa_circ_0013809	3.267	0.001	TCONS_ 00001137	chr1	1.43E+08	1.43E+08	+	10928	10928
hsa_circ_0010146	3.009	5E-04	FBXO42	chr1	16632297	16641930	-	9633	384
hsa_circ_0011287	2.774	0.001	SERINC2	chr1	31901824	31902385	+	561	233
hsa_circ_0012451	2.634	0.012	EPS15	chr1	51926762	51938620	-	11858	486
hsa_circ_0014700	2.606	3E-04	TMEM79	chr1	1.56E+08	1.56E+08	+	9531	2226
hsa_circ_0040024	2.557	0.005	CIRH1A	chr16	69184439	69201745	+	17306	1863
hsa_circ_0011748	2.488	0.013	SF3A3	chr1	38445136	38447483	-	2347	291
hsa_circ_0014566	2.485	3E-04	ASH1L	chr1	1.55E+08	1.55E+08	-	5044	571
hsa_circ_0014940	2.39	0.006	NCSTN	chr1	1.6E+08	1.6E+08	+	2658	682
hsa_circ_0014387	2.342	0.002	UBAP2L	chr1	1.54E+08	1.54E+08	+	199	199
hsa_circ_0011744	2.328	0.011	SF3A3	chr1	38435025	38450451	-	15426	978
hsa_circ_0012513	2.328	0.008	ORC1	chr1	52838500	52847433	-	8933	925
hsa_circ_0011408	2.189	3E-04	HDAC1	chr1	32797074	32799224	+	2150	1049
hsa_circ_0014917	2.133	0.004	COPA	chr1	1.6E+08	1.6E+08	-	13339	537
hsa_circ_0011707	2.079	0.01	GNL2	chr1	38049467	38059447	-	9980	572
hsa_circ_0013510	2.063	0.003	WDR77	chr1	1.12E+08	1.12E+08	-	5120	426
hsa_circ_0013164	2.001	3E-04	EVI5	chr1	93029198	93170301	-	141103	1837
hsa_circ_0011555	2.001	8E-04	ZMYM4	chr1	35854510	35855699	+	1189	324
hsa_circ_0010492	1.979	0.021	EIF4G3	chr1	21377358	21377487	-	129	129
hsa_circ_0014650	1.977	6E-04	ARHGEF2	chr1	1.56E+08	1.56E+08	-	684	247
hsa_circ_0001067	1.95	3E-04	UGGT1	chr2	1.29E+08	1.29E+08	+	18451	1316
hsa_circ_0011145	1.94	9E-04	SNHG3	chr1	28832731	28837404	+	4673	2204
hsa_circ_0013948	1.919	0.005	CHD1L	chr1	1.47E+08	1.47E+08	+	13070	738
hsa_circ_0014565	1.915	5E-05	ASH1L	chr1	1.55E+08	1.55E+08	-	89527	2335
hsa_circ_0013290	1.893	0.002	AGL	chr1	1E+08	1E+08	+	7429	947
hsa_circ_0012061	1.833	0.002	SZT2	chr1	43896619	43912839	+	16220	4512
hsa_circ_0011967	1.807	0.007	PPIH	chr1	43124514	43133568	+	9054	946
hsa_circ_0001506	1.783	0.005	SERINC5	chr5	79505531	79506139	-	608	608
hsa_circ_0013598	1.767	1E-05	PHTF1	chr1	1.14E+08	1.14E+08	-	156	156
hsa_circ_0040071	1.766	2E-04	NFAT5	chr16	69602397	69729282	+	126885	4647
hsa_circ_0000166	1.76	0.029	C1orf27	chr1	1.86E+08	1.86E+08	+	12995	366
hsa_circ_0012844	1.759	0.008	CACHD1	chr1	65016276	65068595	+	52319	319
hsa_circ_0013038	1.756	0.001	FUBP1	chr1	78429746	78433350	-	3604	791
hsa_circ_0013999	1.737	0.024	ANP32E	chr1	1.5E+08	1.5E+08	-	5325	627

hsa_circ_0012482	1.717	0.005	NRD1	chr1	52299688	52299842	-	154	154
hsa_circ_0011740	1.714	0.007	INPP5B	chr1	38397584	38406470	-	8886	252
hsa_circ_0011144	1.711	8E-04	SNHG3	chr1	28832454	28837404	+	4950	2346
hsa_circ_0014103	1.704	0.002	SEMA6C	chr1	1.51E+08	1.51E+08	-	89	89
hsa_circ_0040008	1.698	0.004	TMCO7	chr16	69007930	69074324	+	66394	407
hsa_circ_0014326	1.683	0.01	DENND4B	chr1	1.54E+08	1.54E+08	-	9253	2813
hsa_circ_0012034	1.68	0.003	SZT2	chr1	43868847	43892209	+	23362	3403
hsa_circ_0010533	1.667	3E-04	USP48	chr1	22021558	22021714	-	156	156
hsa_circ_0011031	1.648	7E-04	GPATCH3	chr1	27220726	27220901	-	175	175
hsa_circ_0011164	1.632	2E-04	YTHDF2	chr1	29063135	29096287	+	33152	3060
hsa_circ_0000585	1.593	0.037	SNORD116-19	chr15	25328542	25339104	+	10562	10562
hsa_circ_0014595	1.593	2E-04	MSTO1	chr1	1.56E+08	1.56E+08	+	135	135
hsa_circ_0013110	1.592	0.002	CCBL2	chr1	89434352	89454034	-	19682	304
hsa_circ_0014760	1.583	0.035	IQGAP3	chr1	1.57E+08	1.57E+08	-	14318	1335
hsa_circ_0001594	1.577	0.031	HIST1H2AJ	chr6	27775963	27782476	-	6513	6513
hsa_circ_0001175	1.569	0.035	YTHDF1	chr20	61826924	61827836	-	912	912
hsa_circ_0010367	1.568	7E-05	UBR4	chr1	19503085	19503221	-	136	136
hsa_circ_0014762	1.564	0.001	IQGAP3	chr1	1.57E+08	1.57E+08	-	4132	535
hsa_circ_0011873	1.559	0.002	CAP1	chr1	40506254	40538321	+	32067	2773
hsa_circ_0010177	1.549	0.012	ATP13A2	chr1	17312452	17322795	-	10343	2500
hsa_circ_0015038	1.549	0.004	NOS1AP	chr1	1.62E+08	1.62E+08	+	14837	3482
hsa_circ_0013348	1.539	8E-04	SLC30A7	chr1	1.01E+08	1.01E+08	+	10779	546
hsa_circ_0013410	1.528	0.031	TMEM167B	chr1	1.1E+08	1.1E+08	+	6152	2750
hsa_circ_0014640	1.526	8E-04	KIAA0907	chr1	1.56E+08	1.56E+08	-	12475	1228
hsa_circ_0014499	1.505	4E-04	THBS3	chr1	1.55E+08	1.55E+08	-	108	108
hsa_circ_0040029	1.49	0.048	CIRH1A	chr16	69189773	69197081	+	7308	483
hsa_circ_0001108	1.487	0.028	SP140L	chr2	2.31E+08	2.31E+08	+	81486	41507
hsa_circ_0012972	1.483	0.002	PIGK	chr1	77554666	77620306	-	65640	3767
hsa_circ_0014475	1.483	3E-04	DCST2	chr1	1.55E+08	1.55E+08	-	2244	400
hsa_circ_0011141	1.479	0.004	PHACTR4	chr1	28802618	28819603	+	16985	672
hsa_circ_0011695	1.455	0.042	LSM10	chr1	36859030	36863493	-	4463	850
hsa_circ_0011655	1.444	0.031	EIF2C3	chr1	36505390	36520746	+	15356	632
hsa_circ_0040064	1.438	0.016	TERF2	chr16	69404111	69406258	-	2147	508
hsa_circ_0013102	1.428	0.044	PKN2	chr1	89237103	89237562	+	459	363
hsa_circ_0013808	1.41	0.018	TCONS_l2_00002628	chr1	1.43E+08	1.43E+08	+	169	169
hsa_circ_0010604	1.407	0.027	HSPG2	chr1	22159959	22201026	-	41067	7450
hsa_circ_0012220	1.399	0.023	TESK2	chr1	45820974	45956840	-	135866	943

hsa_circ_0012954	1.392	0.017	ZRANB2	chr1	71536509	71536679	-	170	170
hsa_circ_0014872	1.391	0.032	DCAF8	chr1	1.6E+08	1.6E+08	-	1222	239
hsa_circ_0014268	1.387	0.023	ILF2	chr1	1.54E+08	1.54E+08	-	7789	887
hsa_circ_0013307	1.38	0.011	SASS6	chr1	1.01E+08	1.01E+08	-	7529	1005
hsa_circ_0040070	1.357	0.015	NFAT5	chr16	69602397	69693802	+	91405	1358
hsa_circ_0010445	1.353	2E-04	HP1BP3	chr1	21091869	21107033	-	15164	990
hsa_circ_0040048	1.352	0.002	VPS4A	chr16	69350127	69358946	+	8819	1915
hsa_circ_0011275	1.313	0.001	SERINC2	chr1	31885962	31907527	+	21565	1999
hsa_circ_0012745	1.306	0.009	FGGY	chr1	59762624	59844509	+	81885	766
hsa_circ_0010033	1.305	0.01	FHAD1	chr1	15708523	15719713	+	11190	11190
hsa_circ_0015037	1.292	2E-04	ATF6	chr1	1.62E+08	1.62E+08	+	71	71
hsa_circ_0011777	1.226	0.002	MACF1	chr1	39748860	39793025	+	44165	3919
hsa_circ_0011233	1.221	0.006	PUM1	chr1	31422979	31426828	-	3849	527
hsa_circ_0002044	1.218	8E-04	MTHFD1L	chr6	1.51E+08	1.51E+08	+	2973	226
hsa_circ_0010737	1.215	0.006	HSPG2	chr1	22188249	22205614	-	17365	2612
hsa_circ_0013417	1.206	0.019	CELSR2	chr1	1.1E+08	1.1E+08	+	10210	3745
hsa_circ_0011877	1.205	0.049	CAP1	chr1	40531886	40535546	+	3660	469
hsa_circ_0001379	1.193	0.027	TFRC	chr3	1.96E+08	1.96E+08	-	147	147
hsa_circ_0013998	1.191	0.011	ANP32E	chr1	1.5E+08	1.5E+08	-	8753	682
hsa_circ_0014165	1.187	0.032	PSMB4	chr1	1.51E+08	1.51E+08	+	120	120
hsa_circ_0010571	1.176	0.021	HSPG2	chr1	22157474	22176685	-	19211	4377
hsa_circ_0013056	1.174	0.042	CTBS	chr1	85028939	85029101	-	162	162
hsa_circ_0013408	1.172	0.029	WDR47	chr1	1.1E+08	1.1E+08	-	803	803
hsa_circ_0014873	1.152	0.045	DCAF8	chr1	1.6E+08	1.6E+08	-	60710	24444
hsa_circ_0012021	1.135	0.013	CDC20	chr1	43825639	43828873	+	3234	1146
hsa_circ_0010836	1.134	0.023	KDM1A	chr1	23397717	23408066	+	10349	885
hsa_circ_0014620	1.133	0.001	GON4L	chr1	1.56E+08	1.56E+08	-	26910	3609
hsa_circ_0010191	1.126	0.003	RCC2	chr1	17736469	17749332	-	12863	941
hsa_circ_0011356	1.123	0.02	TXLNA	chr1	32645344	32658031	+	12687	1148
hsa_circ_0013380	1.115	0.004	FNDC7	chr1	1.09E+08	1.09E+08	+	5725	1029
hsa_circ_0013401	1.114	0.001	CLCC1	chr1	1.09E+08	1.09E+08	-	2838	350
hsa_circ_0012205	1.112	0.03	HECTD3	chr1	45470271	45473993	-	3722	904
hsa_circ_0010542	1.108	0.019	HSPG2	chr1	22148736	22188608	-	39872	9508
hsa_circ_0012002	1.102	0.007	SLC2A1	chr1	43394880	43395706	-	826	456
hsa_circ_0012424	1.099	0.011	EPS15	chr1	51860052	51887793	-	27741	1342
hsa_circ_0015052	1.081	0.018	UAP1	chr1	1.63E+08	1.63E+08	+	189	189
hsa_circ_0014433	1.07	0.007	ADAR	chr1	1.55E+08	1.55E+08	-	12760	1658
hsa_circ_0013111	1.065	0.001	RBMXL1	chr1	89445138	89458643	-	13505	5087

hsa_circ_0001401	1.063	0.026	TBC1D1	chr4	38022211	38029500	+	7289	330
hsa_circ_0011996	1.059	0.05	C1orf50	chr1	43239233	43240539	+	1306	219
hsa_circ_0012598	1.055	0.023	TMEM48	chr1	54238001	54254869	-	16868	326
hsa_circ_0014540	1.033	0.017	FDPS	chr1	1.55E+08	1.55E+08	+	1073	286
hsa_circ_0010375	1.025	0.01	UBR4	chr1	19518974	19520051	-	1077	185
hsa_circ_0014939	1.006	0.003	NCSTN	chr1	1.6E+08	1.6E+08	+	122	122
hsa_circ_0014226	-1.05	0.044	S100A6	chr1	1.54E+08	1.54E+08	-	1642	683
hsa_circ_0012535	-1.05	0.041	ZCCHC11	chr1	52903891	52927288	-	23397	811
hsa_circ_0011900	-1.05	0.031	SMAP2	chr1	40874324	40875507	+	1183	165
hsa_circ_0010546	-1.05	0.028	HSPG2	chr1	22151029	22160107	-	9078	1914
hsa_circ_0010775	-1.06	0.002	HSPG2	chr1	22211812	22213827	-	2015	252
hsa_circ_0012862	-1.06	0.016	JAK1	chr1	65301078	65330655	-	29577	2379
hsa_circ_0012442	-1.25	0.025	EPS15	chr1	51875206	51913807	-	38601	714
hsa_circ_0013455	-1.27	0.019	PSMA5	chr1	1.1E+08	1.1E+08	-	16532	674
hsa_circ_0011689	-1.35	0.048	STK40	chr1	36814300	36824423	-	10123	627
hsa_circ_0011120	-1.39	0.026	EYA3	chr1	28362054	28369161	-	7107	284
hsa_circ_0013454	-1.42	0.017	PSMA5	chr1	1.1E+08	1.1E+08	-	5436	552
hsa_circ_0014348	-1.43	0.005	CREB3L4	chr1	1.54E+08	1.54E+08	+	1222	835
hsa_circ_0011213	-1.45	0.042	PUM1	chr1	31404352	31418330	-	13978	2416
hsa_circ_0010395	-1.5	0.024	MRTO4	chr1	19583557	19586622	+	3065	1827
hsa_circ_0012226	-1.57	0.041	AKR1A1	chr1	46018107	46018235	+	128	128
hsa_circ_0010777	-1.67	9E-04	HSPG2	chr1	22216473	22216978	-	505	220
hsa_circ_0010832	-1.69	0.02	KDM1A	chr1	23395031	23410184	+	15153	1779
hsa_circ_0011969	-1.77	0.01	PPIH	chr1	43124898	43142429	+	17531	599
hsa_circ_0000031	-1.83	0.034	RPL11	chr1	24019108	24020403	+	1295	248
hsa_circ_0014161	-1.84	0.014	PSMB4	chr1	1.51E+08	1.51E+08	+	592	199
hsa_circ_0012171	-1.99	0.047	RPS8	chr1	45241245	45243461	+	2216	410
hsa_circ_0000104	-2.18	0.036	CD53	chr1	1.11E+08	1.11E+08	+	266	266
